# Systemic inflammation is a risk factor for oral health: an analysis of data from the UK Biobank

**DOI:** 10.3389/fimmu.2025.1651947

**Published:** 2026-01-22

**Authors:** Martin Berger, Jaana-Sophia Kern, Ulrike Schulze-Späte, Stefan Wolfart, Joachim Jankowski, Michael Wolf, Nikolaus Marx, Katharina Marx-Schütt

**Affiliations:** 1 Department of Internal Medicine I, University Hospital Aachen, Rheinisch-Westfälische Technische Hochschule (RWTH) University Hospital Aachen, Aachen, Germany; 2 Department of Prosthodontics and Biomaterials, Center for Implantology, RWTH University Hospital Aachen, Aachen, Germany; 3 Section of Geriodontics, Department of Conservative Dentistry and Periodontology, Center of Dental Medicine, University Hospital Jena, Jena, Germany; 4 Institute for Molecular Cardiovascular Research, RWTH University Hospital Aachen, Aachen, Germany; 5 Department of Orthodontics, RWTH University Hospital Aachen, Aachen, Germany

**Keywords:** C reactive protein, inflammation, interleukein-6, oral-general health, single nucleotide polymorphisms

## Abstract

**Background:**

Oral health has been implicated as a contributor to systemic inflammation and cardiovascular disease. However, the reverse relationship—whether systemic inflammation causally affects oral health—remains poorly defined. This study investigates the directional role of systemic inflammation in oral disease using genetic and phenotypic data from the UK Biobank.

**Methods:**

We analyzed data from 468,460 UK Biobank participants, integrating self-reported oral health measures with plasma levels of high-sensitivity C-reactive protein (hsCRP). To assess causality, we examined the IL6R single nucleotide polymorphism rs2228145 (p.Asp358Ala), a well-characterized variant associated with impaired IL-6 receptor signaling and reduced systemic inflammation.

**Results:**

Higher hsCRP tertiles were significantly associated with increased prevalence of poor oral health indicators, including tooth loss, dentures, bleeding gums, and loose teeth (all p < 0.001). Carriers of the IL6R C/C genotype exhibited significantly lower hsCRP levels and a reduced burden of oral pathology, including lower odds for toothache (OR 0.91 [95%CI 0.87-0.94]), bleeding gums (OR 0.97 [95%CI 0.94-0.99]), and loose teeth (OR 0.92 [95%CI 0.88-0.96]).

**Conclusion:**

Our findings support systemic inflammation—mediated via IL-6 signaling—as a causal determinant of impaired oral health. This study provides novel evidence for a directional link from systemic inflammation to oral disease, with potential implications for targeted immunomodulatory interventions in oral health.

## Introduction

Oral health and periodontal inflammation have repeatedly been identified as contributors to cardiovascular and inflammatory disease in epidemiological studies and interventional trials evaluating surrogate parameters (1). Conversely, there is evidence that systemic diseases such as type 2 diabetes can promote periodontal inflammation, indicating a bidirectional relationship (2, 3). Interestingly, while the majority of studies have examined how oral conditions may influence systemic inflammation, the potential contribution of generalized, low-grade systemic inflammation to oral health has received relatively little attention. Importantly, the relationship between systemic inflammation and oral health has not been explored in large-scale genomic studies, although genetic approaches may help to clarify whether observed associations are consistent with a directional influence. Accordingly, this study aimed to investigate whether genetic variants that diminish IL-6 signaling and thereby lower systemic inflammatory activity are associated with improved oral health. To this end we used a genotype-based approach focusing on a well-characterized functional single-nucleotide polymorphism (SNPs) in the IL-6 receptor pathway.

## Methods

Data from 468,460 participants of the UK Biobank with available information on self-reported oral health indicators and high-sensitivity C-reactive protein (hsCRP) plasma levels were analysed (4). Self-reported oral health indicators included tooth loss, denture use, bleeding gums, loose teeth, mouth ulcers and toothache (i.e. summarized as oral health indicators). Of note, these questionnaire-based indicators do not reflect formal clinical diagnoses but represent participants’ subjective experience of oral health burden. The questionnaire was completed during the baseline assessment of each participant. In addition, the genetic IL6 receptor variant p.Asp358Ala (rs2228145), which has been demonstrated to be linked to impaired IL-6 receptor signaling and reduced subclinical inflammation as measured by hsCRP was used to examine whether genetically influenced differences in inflammatory tone are accompanied by differences in self-reported oral health indicators (5). Logistic regression models were adjusted for age, sex, body mass index (BMI), hsCRP, smoking status, and socio-economic indicators (including income and educational attainment). Model residuals fulfilled assumptions of normality. Use of data for this study was approved by the UK Biobank (application number 88924).

## Results

**Figure 1:**
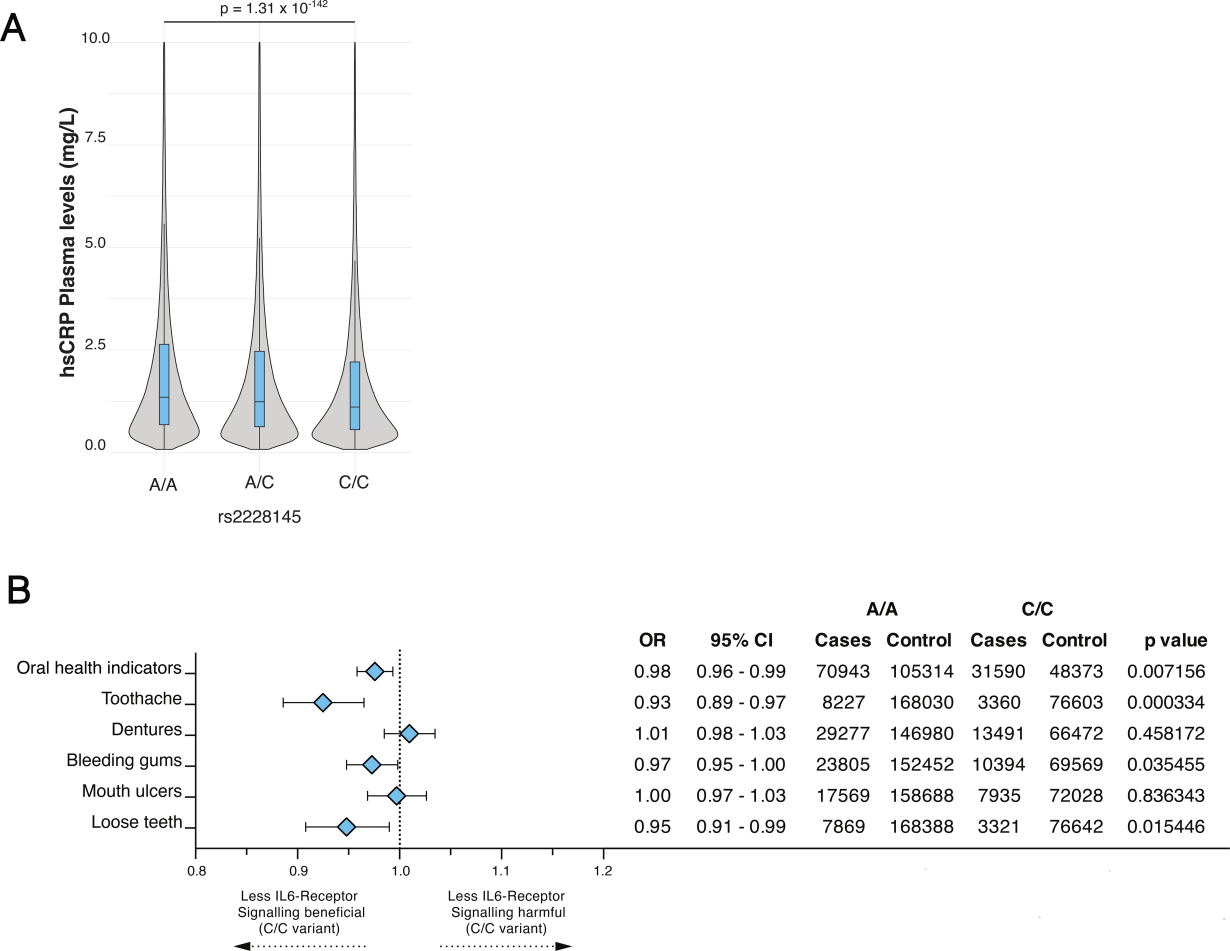
Association between systemic inflammation, IL6-receptor genotype, and perceived oral health **(A)** Clinical and demographic characteristics of 468,460 participants stratified by tertiles of high-sensitivity C-reactive protein (hsCRP) levels. **(B)** Forest plot for the genetic association between IL6-R rs2228145 genotype and various indicators of oral health. Odds ratios (ORs) and 95% confidence intervals (CIs) compare C/C homozygotes to A/A homozygotes. All analysis were adjusted for age, sex, body mass index (BMI), high sensitive CRP (hsCRP) smoking, socioeconomic status (incl. education and income level) and alcohol consumption.

Stratification of hsCRP plasma levels into tertiles (hsCRP_1st_: 0.49mg/L [0.08-0.84] hsCRP_2nd_: 1.33mg/L [0.84-2.11] hsCRP_3rd_: 3.85mg/L [2.11-79.96]) demonstrated expected associations between systemic inflammatory state and cardiovascular disease (all p < 0.001; **Table 1**). Higher hsCRP tertiles were also associated with a greater prevalence of adverse self-reported oral health indicators (1^st^ vs. 3^rd^: 35% vs. 45%; p < 0.001) including denture use (1^st^ vs. 3^rd^: 12% vs. 21%; p < 0.001) and loose teeth (1^st^ vs. 3^rd^: 3.2% vs. 5.6%; p < 0.001; **Table 1**). To explore whether genetically influenced differences in IL-6 signaling show patterns consistent with these associations, we examined the SNP p.Asp358Ala (rs2228145) linked to impaired IL-6 signaling (5). Carriers of the C/C variant, exhibited a 18% decrease in hsCRP levels compared to carriers of the A/A variant (A/A: 2.77±4.52 mg/L vs. C/C: 2.29±4.00mg/L; p < 0.001; **Figure 1A**). Furthermore, logistic regression indicated that presence of the C/C variant was associated with lower odds of the self-reported oral health composite endpoint (OR 0.98, 95%CI 0.96–0.99, p=0.007). Exploratory endpoint analyses showed reduced odds for toothache (OR 0.93, 95%CI 0.89–0.97, p=0.0003), bleeding gums (OR 0.97, 95%CI 0.95–1.00, p=0.035), and loose teeth (OR 0.95, 95%CI 0.91–0.99, p=0.015), whereas associations with denture use (OR 1.01, 95%CI 0.91–0.99, p=0.45) and mouth ulcers (OR 1.00, 95%CI 0.97–1.03, p=0.83) were neutral (**Figure 1B**).

**Table 1:** Clinical and demographic characteristics of 468,460 participants stratified by tertiles of high-sensitivity C-reactive protein (hsCRP) levels.

High Sensitive CRP Tertiles					
Characteristic	Overall	1st	2nd	3rd	p-value
	N = 468,460	N = 156,154	N = 156,153	N = 156,153	
hsCRP (mg/L)	2.60 (4.36)	0.49 (0.0–0.8)	1.33 (0.8–2.1)	3.85 (2.1–79.9)	<0.001
Age (years)	57 (8)	55 (8)	57 (8)	57 (8)	<0.001
Sex (male)	214,345 (46%)	71,922 (46%)	76,076 (49%)	66,347 (42%)	<0.001
Medical History					
Diabetes	20,128 (4.3%)	4,762 (3.0%)	6,329 (4.1%)	9,037 (5.8%)	<0.001
Heart failure	15,998 (3.4%)	3,203 (2.1%)	4,871 (3.1%)	7,924 (5.1%)	<0.001
Hypertension	126,474 (27%)	30,364 (19%)	42,275 (27%)	53,835 (34%)	<0.001
Myocardial infarction	10,759 (2.3%)	3,091 (2.0%)	3,615 (2.3%)	4,053 (2.6%)	<0.001
Stroke	7,082 (1.5%)	1,814 (1.2%)	2,236 (1.4%)	3,032 (1.9%)	<0.001
Medical Intervention					
PCI	4,890 (1.0%)	1,610 (1.0%)	1,639 (1.0%)	1,641 (1.1%)	0.83
Bypass graft surgery	3,511 (0.7%)	1,113 (0.7%)	1,175 (0.8%)	1,223 (0.8%)	0.073
Dental Status					
Comp Oral Health Indicators	187,176 (40%)	54,786 (35%)	61,802 (40%)	70,588 (45%)	<0.001
Toothache	20,718 (4.4%)	6,263 (4.0%)	6,696 (4.3%)	7,759 (5.0%)	<0.001
Dentures	78,168 (17%)	18,839 (12%)	25,885 (17%)	33,444 (21%)	<0.001
Bleeding gums	62,249 (13%)	19,887 (13%)	20,442 (13%)	21,920 (14%)	<0.001
Painful gums	14,249 (3.0%)	4,041 (2.6%)	4,527 (2.9%)	5,681 (3.6%)	<0.001
Loose teeth	20,312 (4.3%)	4,922 (3.2%)	6,589 (4.2%)	8,801 (5.6%)	<0.001
Mouth ulcers	47,156 (10%)	15,695 (10%)	15,392 (9.9%)	16,069 (10%)	<0.001

## Discussion

In a large-scale population analysis from the UK Biobank, we show that genetically determined attenuation of IL-6 signaling via the IL6R p.Asp358Ala variant is associated with improved self-reported composite indices of oral health. These data suggest that systemic inflammation may be a driver of oral pathologies and challenge the prevailing concept that poor oral health contributes unidirectionally to systemic inflammation. Further, they provide a framework for future studies that employ clinically validated oral health outcomes, longitudinal designs, and formal causal inference approaches to clarify underlying mechanisms and determine their potential clinical relevance.

A key limitation of our work is the reliance on self-reported oral health data in the UK Biobank. While such measures cannot replace clinical diagnoses, selected items—particularly tooth loss, loose teeth, and gingival bleeding—have demonstrated acceptable validity for population-level surveillance of periodontitis (6, 7). Nevertheless, oral health indices in our study represent general oral health burden, recognizing that items such as toothache, denture use, or mouth ulcers may arise from heterogeneous or non-periodontal conditions. While these limitations apply, the aim of this manuscript was to focus on the association between oral health and inflammation; phenotypic precision, disease staging, and subclinical characterization lie beyond its scope. In addition, although the IL6R p.Asp358Ala variant is known to attenuate IL-6 signaling, it concomitantly increases circulating soluble IL-6, potentially eliciting pleiotropic downstream effects that were not assessed in the present analysis.

In conclusion, here we demonstrate that the genetic variant IL6R p.Asp358Ala is associated with improved self-reported composite indices of oral health and implies a bidirectional relationship between oral pathologies and inflammation.

## Data Availability

The data used in this study were obtained from UK Biobank under a material transfer and data access agreement. In accordance with UK Biobank’s governance framework, researchers are not permitted to share individual-level UK Biobank data with third parties. Access to the data is restricted to approved researchers and specific approved projects, and all secondary use requires a separate application directly to UK Biobank. Requests to access these datasets should be directed to the corresponding author.
